# The impact of speech rate on sentence recognition by elderly individuals

**DOI:** 10.5935/1808-8694.20130136

**Published:** 2015-10-08

**Authors:** Alexandre Hundertmarck Lessa, Maristela Julio Costa

**Affiliations:** a MSc. in Human Communications Disorders, Federal University of Santa Maria (Ph.D Student, Human Communication Disorders, Federal University of Santa Maria; Scholarship Holder, FAPERGS/CAPES); b PhD in Human Communication Disorder Sciences, Federal University of São Paulo (Adjunct Professor, Department of Speech and Hearing Therapy, Federal University of Santa Maria; Research Scholarship Holder - Level 2, CNPq). Federal University of Santa Maria - UFSM

**Keywords:** auditory perception, elderly, hearing, speech discrimination tests, speech perception

## Abstract

Difficulty understanding speech, particularly in situations unfavorable to communication, is a common complaint among elderly individuals.

**Objective:**

To verify the variables connected to hearing loss and stimulus presentation rate and their impact on the speech recognition skills of elderly subjects in quiet and noisy environments.

**Method:**

This case-control study included two groups of subjects (31 elderly subjects with normal hearing and 26 with hearing loss) exposed to the List of Sentences in Portuguese and the Slowed List of Sentences in Portuguese tests. Sentence recognition indices were calculated for tests done against noisy and quiet backgrounds at a normal and reduced speech rate. Data sets were submitted to statistical analysis.

**Results:**

elderly subjects from both groups had better test results when sentences were played at a slower rate. Statistically significant difference was seen for both groups when the tests were carried out on a quiet background and for the group with hearing loss when tested on a noisy background.

**Conclusion:**

regardless of their peripheral hearing, the elderly subjects included in this study were more able to recognize speech when sentences were played at a slower rate against a quiet background. When sentences were played against a noisy background, the elderly subjects with hearing loss had more significant performance improvements than the ones with normal hearing when sentences were played at a slower rate.

## INTRODUCTION

Interest has grown around the issues connected to aging and temporal auditory processing. Elderly subjects without hearing loss have reported difficulty detecting lower intensity sound stimuli and understanding speech, particularly in situations in which competing sound stimuli are present, while others with hearing loss sometimes fail to present similar complaints^1^.

The rapid sequence of connected sound stimuli characteristically seen in speech may be overwhelming to subjects with impaired temporal auditory processing, who end up missing brief but relevant bits of acoustic information required for effective communication to occur^2^. In addition to hearing loss, diminished temporal auditory processing capability is believed to be one of the factors involved in the difficulty understanding speech experienced by elderly individuals^2^.

Difficulty understanding speech is a common complaint among elderly subjects, particularly in contexts unfavorable to communication, such as when speech is delivered at a faster rate3., 4.. Speech recognition in noise4., 5., 6. also ranks as one of the most significant barriers to communication faced by the elderly.

Communication deficits have been reported by elderly individuals with and without hearing loss^4^. However, evidence indicates that speech recognition performance may be diminished in elderly individuals with hearing loss^7^.

Traditional tests used to evaluate temporal auditory processing skills resort to non-verbal stimuli; however, these tests are known for not completely assessing one's ability to understand speech. Sentence-based speech recognition tests, by their turn, closely emulate situations of everyday life^8^. The joint application of the List of Sentences in Portuguese - LSP^9^ and the Slowed List of Sentences in Portuguese tests - SLSP^10^ can be performed to investigate sentence recognition skills in the absence and presence of noise. The comparison between the results of both tests also allows the production of inferences on temporal auditory function.

This study aimed to determine the influence of variables hearing loss and speech rate in the elderly, against quiet and noisy backgrounds.

## METHOD

This study is linked to project ‘Sentence recognition at different speech rates’ registered in the Project Department of the Center for Health Sciences under n^o^. 029 457. The study was approved by the Research Ethics Committee of an institution of higher education and given permit 0098.0.243.000–11. The study was carried out in the Laboratory for Hearing Aids of the Speech and Hearing Therapy Service of a higher education institution.

All subjects were informed of the objectives, rationale, benefits, risks, and research procedures; participants were given access to ample clarification; participant identities and data were guarded by the researcher and treated confidentially; participants were given permission to leave the study at any time for any reason; participants were offered direct access to the examiner in person or by phone when they thought necessary.

Data was contained in the informed consent term, which was duly read and signed by those who agreed to participate in the study.

The group of elderly subjects with normal hearing (control group) was made up of individuals coming from associations of seniors, while the group of subjects with hearing loss (case group) had elderly citizens awaiting for hearing aids granted by a federal program managed by the Institution and some individuals from associations of seniors.

The enrollment criteria included: age over 60 years (the World Health Organization's definition for elderly subjects in developing countries); having a Percentage Speech Recognition Index (PSRI) of 72% or above; no outer ear involvement; no history of alterations or deficits that could compromise the execution of the test procedures (neurological, psychological, mental or cognitive involvement); and/or noticeable speech disorders.

Additionally, subjects in the control group had to have auditory thresholds within normal limits - tritone average equal to or less than 25 dB^11^. Individuals in the case group had to have mild to moderately severe sensorineural hearing loss^11^ without ever having worn hearing aids.

### Sample selection

Before they were divided into groups, the volunteers were interviewed, had their outer ear canals inspected, underwent pure-tone audiometry (PTA) at 250–8000 Hz for air conduction and 500–4000 Hz for bone conduction, had their Speech Recognition Thresholds (SRT) determined for disyllables, and their Percent Speech Recognition Indices (PSRI) for monosyllables in a soundproof booth, using a two-channel digital audiometer (Interacoustics Affinity AC440) and Telephonics TDH-39P earphones.

### Composition of groups

The group of normal hearing elderly (control group) consisted of 31 subjects, six males and 25 females, aged between 61 and 81 years. The group of elderly people with hearing loss (case group) had 26 subjects, 12 males and 15 females, aged 60 to 84 years. Fourteen subjects in the case group had mild sensorineural hearing loss in the better ear and 12 had moderate sensorineural hearing loss.

### Data collection

After the subjects were split in groups, their Percent Sentence Recognition Indices in Silence (PSRIS) and Percent Sentence Recognition Indices in Noise (PSRIN) were obtained through the application of the List of Sentences in Portuguese - LSP^9^; Speech Recognition Thresholds of Slowed Sentences in Silence (SRTSSS), Percent Sentence Recognition Indices of Slowed Sentences in Silence (PSRISSS), Speech Recognition Thresholds of Slowed Sentences in Noise (SRTSSN), Percent Sentence Recognition Indices of Slowed Sentences in Noise (PSRISSN) were obtained through the application of the Slowed List of Sentences in Portuguese test - SLSP^10^.

The LSP^9^ test consists of a book and a Compact Disc featuring a list of 25 sentences in Brazilian Portuguese, called List 1A^12^, seven lists of 10 sentences each, from 1B to 7B^13^, noise in the spectrum of speech^14^, and a pure tone used for calibration.

The SLSP^10^ is a development from the LSP^9^. It includes the same sentences, modified by professionals from the areas of physics, electrical engineering and acoustic engineering. Changes were made so that all sentences were 25% longer and the spectral content of the materials were minimally altered. An algorithm was used to introduce a pattern of modification that does not generate musical-subjective alteration. This algorithm is included in Steinberg/Yamaha's software Cubase SX/SL 3.

A new CD was produced to contain the eight tracks with the list of sentences from the original LSP, however with speech rate decreased beyond the range of the calibration pure tone, and background noise within the spectrum of speech included in the original CD^9^.

Test measurements were made in a soundproof booth with the two-channel digital audiometer described above, in addition to an amplifier equipped with Iridium PA100 speakers for free field measurements.

The calibration of the equipment for free field measurements was carried out on the place where the patients would be positioned, i.e., one meter away from the speakers at 0°, 0° azimuth, by a trained expert registered at Inmetro São Paulo. Sound pressure level (SPL) measurements were made using the meter's fast-response A scale, as it is the one that most closely matches the human auditory response, in addition to being used by most researchers in this area.

Open field measurements were monitored throughout the study by the examiner with the aid of a RadioShack digital sound pressure meter, considering the characteristics of the test signal and the need to always maintain the same acoustic conditions in the environment.

The reference pure tone present in the first track of the CD was used to establish the calibration parameters of the channel in which the sentences were played, thus ensuring consistent speech stimuli reproduction.

Noise was used as the reference in the calibration of the noise present in the other channel of the CD, as it is a continuous sound. The output of each channel was calibrated using the audiometer's VU-meter. The pure tone in channel one and the noise in channel two were set at zero.

The sentences and noise recorded on the CD in independent channels were played on a Toshiba 4149 CD player coupled to the described audiometer and speakers.

### Sentence Recognition measurement acquisition

Measurements were made in the following order: SRTSSS, PSRISSS, PSRIS, SRTSSN, PSRISSN, and PSRIN. Patients were trained to become familiarized with the test protocol before measurements were made.

### Training

Sentences 1 to 5 on slowed sentence list 1A were played against a quiet background to capture SRTSSS, PSRISSS and PSRIS.

In addition to familiarizing subjects with the test, training also served to determine the necessary initial intensity of sentence presentation so that the subjects could understand the first sentence on each of the test lists^15^. The test was repeated, and then the measurements against a background of noise at 65 dB SPL (A) were made, this time with sentences 6 to 10 on list 1A, to capture SRTSSN, PSRISSN, and PSRIN.

### Sentence recognition threshold acquisition

Speech Recognition Thresholds of Slowed Sentences in Silence (SRTSSS) were determined using list 1B from the SLSP against a quiet background. Speech Recognition Thresholds of Slowed Sentences in Noise (SRTSSN) were obtained using list 4B from the SLSP against a background of noise.

A sequential/adaptive or ascending/descending strategy was used in the verification of speech recognition thresholds of slowed sentences^16^. This allows the measurement of the level required for the individual to correctly identify about 50% of the speech stimuli presented in a given signal-to-noise ratio (SNR).

In this strategy, when subjects correctly recognized the speech stimuli, the intensity of presentation of the next sentence was reduced; and when they failed to recognize the speech stimuli, the intensity of presentation was increased. A response was considered correct only when the subject was able to repeat the entire sentence, without error or omissions.

Stimuli were presented in intervals of 4 dB until the first change in the type of response; then the intervals were decreased to 2 dB until all sentences were played, as recommended in the literature^16^.

Noise intensity was kept at a constant 65 dB SPL (A) during the acquisition of SRTSSN.

The acquired thresholds were used to determine the sentence list presentation parameters for the acquisition of percent sentence recognition indices.

### Percent Sentence Recognition Index acquisition

First, the Percent Sentence Recognition Index of Slowed Sentences in Silence (PSRISSS) was determined using list 2B from the set of slowed sentences (SLSP), followed by the Percent Sentence Recognition Index in Silence (PSRIS) assessed using list 3B of sentences played at a normal rate (LSP). The PSRISSN was obtained using list 5B in the SLSP, and the PSRIN was tested using list 6B in the LSP.

The stimulus intensities found in the SRTSSS and SRTSSN tests were used to set up the sentence presentation parameters in the percent sentence recognition index tests in silence and noise, respectively. Noise intensity was kept at a constant 65 dB SPL (A) during the acquisition of SRTSSN and PSRIN.

Indices were calculated based on word scores^17^. This calculation method was chosen for offering a more precise analysis of what the patient is able or not to recognize during a conversation, without neglecting right responses.

In this calculation method, two points are awarded for each content word (nouns, adjectives, verbs, adverbs, and numbers) and one point for each functional word (articles, prepositions, conjunctions, pronouns, and interjections) correctly repeated. At the end of the presentation of the list, the total score is multiplied by a preset factor to yield the percent rate of correct responses, which will make up the individual's Percent Sentence Recognition Index.

### Statistical analysis

The Lilliefors test was used to verify the normality of the variables; the *t*-test was used to analyze the significance of dependent variables with a normal distribution; the Wilcoxon signed-rank test was used to treat variables that did not follow a normal distribution.

A level of statistical significance of 5% was considered (*p* < 0.05).

## RESULTS

In the case group, the values obtained for PSRIS, PSRISSS, PSRIN and PSRISSN had a normal distribution, according to the Lilliefors test. Table 1 shows that subjects in the control group performed better when presented with slowed sentences; statistically significant difference was observed for tests done against a background of silence, but not in noise.

**Table 1:** Percent Sentence Recognition Indices and differences in performance on both tests of subjects in the control group.

Subject/Measurement	PSRIS	PSRISSS	Dif (PSRIS-PSRISSS)	PSRIN	PSRISSN	Dif (PSRIN-PSRISSN)
SC1	58.50%	64.41%	-5.91%	52.17%	61.20%	-9.03%
SC2	63.18%	84.75%	-21.57%	69.93%	51.6%	18.33%
SC3	66.70%	87.01%	-20.31%	57.70%	74.40%	-16.70%
SC4	47.97%	79.10%	-31.13%	63.27%	44.40%	18.87%
SC5	60.84%	79.10%	-18.26%	69.93%	60.00%	9.93%
SC6	57.33%	63.28%	-5.95%	83.25%	86.40%	-3.15%
SC7	76.05%	73.45%	2.60%	81.03%	49.20%	31.83%
SC8	47.97%	55.37%	-7.40%	77.70%	60.00%	17.70%
SC9	65.52%	63.28%	2.24%	56.61%	30.00%	26.61%
SC10	83.07%	80.25%	2.82%	74.37%	84.00%	-9.63%
SC11	80.73%	91.53%	-10.80%	67.71%	49.20%	18.51%
SC12	70.20%	100.00%	-29.80%	52.17%	46.80%	5.37%
SC13	82.49%	57.60%	24.89%	90.48%	86.58%	3.90%
SC14	81.90%	68.93%	12.97%	38.85%	40.80%	-1.95%
SC15	84.75%	81.90%	2.85%	46.62%	50.40%	-3.78%
SC16	67.86%	73.45%	-5.59%	61.05%	62.40%	-1.35%
SC17	76.05%	85.88%	-9.83%	69.93%	88.80%	-18.87%
SC18	81.90%	96.05%	-14.15%	85.47%	78.00%	7.47%
SC19	66.69%	87.36%	-20.67%	35.20%	37.20%	-2.00%
SC20	51.48%	82.49%	-31.01%	72.15%	56.40%	15.75%
SC21	79.56%	84.75%	-5.19%	43.29%	33.60%	9.69%
SC22	65.52%	100.00%	-34.48%	54.39%	44.40%	9.99%
SC23	57.33%	57.63%	-0.30%	44.40%	34.80%	9.60%
SC24	71.37%	61.02%	10.35%	64.38%	67.20%	-2.82%
SC25	52.65%	66.67%	-14.02%	53.28%	51.60%	1.68%
SC26	51.48%	75.71%	-24.23%	57.72%	46.80%	10.92%
SC27	75.71%	79.10%	-3.39%	48.84%	73.20%	-24.36%
SC28	47.97%	56.50%	-8.53%	43.29%	30.00%	13.29%
SC29	70.02%	80.23%	-10.21%	56.61%	50.40%	6.21%
SC30	77.22%	67.80%	9.42%	67.71%	88.80%	-21.09%
SC31	54.99%	59.89%	-4.90%	53.28%	56.40%	-3.12%
			*p* = 0.001690*			*p* = 0.131644

* Statistically significant difference. SC: Subject on Control Group; PSRIS: Percent Sentence Recognition Indices in Silence; PSRISSS: Percent Sentence Recognition Indices of Slowed Sentences in Silence; Dif: Difference; PSRIN: Percent Sentence Recognition Indices in Noise; PSRISSN: Percent Sentence Recognition Indices of Slowed Sentences in Noise; p: significance level (*t*-test for dependent variables).

According to the Lilliefors test, the values seen in PSRIS, PSRISSS, and PSRISSN in the case group followed a normal distribution. Table 2 shows that case group subjects performed statistically better in either noise or silence when sentences were played at a slower speech rate.

**Table 2:** Percent Sentence Recognition Indices and differences in performance on both tests of subjects in the case group.

Subject/Measurement	PSRIS	PSRISSS	Dif (PSRIS-PSRISSS)	PSRIN	PSRISSN	Dif (PSRIS-PSRISSN)
SE1	71.37%	84.75%	-13.38%	55.50%	46.80%	8.70%
SE2	49.14%	72.30%	-23.16%	69.93%	85.20%	-15.27%
SE3	76.05%	87.01%	-10.96%	72.15%	72.00%	0.15%
SE4	38.61%	62.15%	-23.54%	54.39%	74.24%	-19.85%
SE5	64.35%	72.32%	-7.97%	15.54%	43.20%	-27.66%
SE6	77.22%	72.32%	4.90%	71.04%	91.20%	-20.16%
SE7	56.16%	67.80%	-11.64%	64.38%	44.40%	19.98%
SE8	51.48%	39.55%	11.93%	56.61%	81.60%	-24.99%
SE9	60.84%	65.54%	-4.70%	43.29%	58.80%	-15.51%
SE10	74.88%	67.80%	7.08%	48.84%	74.40%	-25.56%
SE11	91.26%	76.84%	14.42%	61.05%	58.80%	2.25%
SE12	66.69%	63.28%	3.41%	62.16%	63.60%	-1.44%
SE13	83.07%	83.62%	-0.55%	72.15%	88.80%	-16.65%
SE14	81.90%	83.62%	-1.72%	38.85%	58.80%	-19.95%
SE15	74.88%	89.27%	-14.39%	63.27%	79.20%	-15.93%
SE16	63.18%	64.41%	-1.23%	33.30%	33.60%	-0.30%
SE17	64.35%	64.41%	-0.06%	45.51%	57.60%	-12.09%
SE18	47.97%	39.55%	8.42%	62.16%	70.80%	-8.64%
SE19	28.08%	67.80%	-39.72%	12.21%	43.20%	-30.99%
SE20	36.27%	64.41%	-28.14%	71.04%	78.00%	-6.96%
SE21	42.12%	53.11%	-10.99%	83.25%	79.20%	4.05%
SE22	65.52%	80.23%	-14.71%	21.09%	42.00%	-20.91%
SE23	79.56%	67.80%	11.76%	65.49%	70.80%	-5.31%
SE24	97.11%	83.62%	13.49%	56.61%	51.60%	5.01%
SE25	69.03%	73.45%	-4.42%	53.28%	48.00%	5.28%
SE26	59.67%	68.93%	-9.26%	17.76%	28.80%	-11.04%
			*p* = 0.047283*			*p* = 0.002030*

* Statistically significant difference. SE: Subjects in the Case Group; PSRIS: Percent Sentence Recognition Indices in Silence; PSRISSS: Percent Sentence Recognition Indices of Slowed Sentences in Silence; Dif: Difference; PSRIN: Percent Sentence Recognition Indices in Noise; PSRISSN: Percent Sentence Recognition Indices of Slowed Sentences in Noise; *p*: Significance level (*t*-test for dependent variables and Wilcoxon).

## DISCUSSION

The interest around variables hearing loss and rate of stimuli in speech recognition in the tests of elderly individuals arose because of claims that time affects one's ability to recognize speech4., 18. and of the observation that elderly subjects with hearing loss19., 20. may have difficulty understanding what is said to them.

Memory and attention deficits and general progressive reductions in brain function are present in most of the issues faced by aging individuals^21^. Additionally, auditory processing impairments and their impact upon speech recognition further contribute to the challenges faced by the elderly^22^.

Accurate interpretation of speech requires focused attention by the listener. Given that cognitive impairments reduce the ability of the elderly to manage and integrate information, fast-paced speech poses additional challenges to the perception of elderly subjects^22^.

In this study, we tried to verify if speech recognition is impaired by peripheral auditory involvement and whether different rates of speech alter the performance of elderly individuals in speech recognition tests.

A protocol comprised by sentence recognition tests was chosen to that end, as it closely emulates communication situations to which the subjects are exposed in their daily lives^8^.

Initially, the analysis of the measurements obtained against a quiet background made it clear that the groups of elderly subjects with normal hearing (control group) and elderly individuals with hearing loss (case group) showed statistically significant improvements in test scores when presented with slowed sentences, which showed that speech recognition in the subjects of both groups was improved when speech was produced at a lower rate.

Our findings were compared against those of studies in which temporal auditory processing was mostly assessed through non-verbal stimuli, due to the difficulty finding studies in which speech stimuli were used to investigate the correlations between temporal auditory processing and speech recognition.

Some authors2., 23. have looked into the influence of temporal auditory processing upon the performance of elderly subjects on tests used to assess temporal ordering and resolution, and found no differences in performance between groups with normal hearing and hearing loss. This finding suggests that aging is a much bigger factor in the decline in performance experienced by the elderly. Our findings also indicated that elderly subjects, regardless of peripheral hearing, performed better when presented with speech at a lower rate against a background of silence.

Evidence that the rate of speech affects the performance of elderly individuals has been reported in a study^24^ in which the subjects had inferior performance when exposed to compressed sentences presented at a faster rate. Our study adds that in addition to speech recognition being deteriorated as the rate of speech increases, it can also be improved when the speech rate is decreased.

The analysis of the measurements carried out against a noisy background showed that control group subjects had no statistically significant differences in performance when test speech rates were changed. Nonetheless, significant improvement was observed in case group individuals performance when slowed sentences were played. This shows that being spoken to slowly is important for the elderly, particularly for individuals with hearing loss.

Our findings show the importance of educating the families of the elderly, particularly those of individuals in need of wearing hearing aids, on the adoption of communication strategies such as speaking more slowly, so their dear ones can engage in fulfilling conversations. The importance of family education in attaining successful communications with the elderly has been reported^25^.

Our results also serve as a warning to health care workers, so that they can modulate their speech rate to meet the needs of elderly patients.

Although the degree of audibility strongly influences speech recognition, some elderly subjects seem to face more difficulties than expected from the analysis of audiometric configurations. One of the difficulties in speech recognition of the elderly derives from the age-related decline in cognitive skills, changes in auditory processing, or the combination of both^19^.

Authors^26^ have reported that, in addition to hearing loss, other changes are observed in the cognitive function of elderly subjects. These changes in the elderly are characterized by slowness, suggesting the existence of transmission deficits in the temporal processing of these individuals.

The results of speech recognition tests in noise showed clear improvements in the performance of elderly subjects with hearing loss as the rate of speech was decreased, even in the absence of expected intonations or visual cues relied on by individuals to better understand what they hear in noisy environments. This shows how decreases in speech rate are really important for the elderly to better understand what they are being told in noisy environments.

Although positive quantitative results in the SLSP do not necessarily reflect positive auditory performance in tested subjects, this test is an additional tool health care workers can use to obtain objective data to advise elderly patients fitted with hearing aids who still experience communication difficulties.

Thus, it is possible to quantify and demonstrate to elderly subjects that it may take more than hearing aids for them to see significant improvements in communication skills.

Guidance on communication strategies will possibly be better understood and internalized by patients and their families and generate interest and greater compliance to auditory rehabilitation programs when special attention is given to temporal auditory processing and the benefits it brings to one's communication ability.

Hearing aids alone cannot address all patient auditory complaints. Good auditory performance is the outcome of a summation of factors involved in the hearing aid selection and fitting process, including auditory temporal processing.

## CONCLUSION

The findings of this study show that elderly subjects, regardless of peripheral hearing, performed better in speech recognition tests when sentences were played at a lower speech rate against a quiet background.

As for speech in noise, the study suggested that elderly individuals with hearing loss had more significant improvements in speech recognition than subjects with normal hearing when sentences were presented at a lower speech rate.
